# Toxic and Microbiological Effects of Iron Oxide and Silver Nanoparticles as Additives on Extended Ram Semen

**DOI:** 10.3390/ani11041011

**Published:** 2021-04-03

**Authors:** Ioannis A. Tsakmakidis, Theodoros Samaras, Sofia Anastasiadou, Athina Basioura, Aikaterini Ntemka, Ilias Michos, Konstantinos Simeonidis, Isidoros Karagiannis, Georgios Tsousis, Mavroeidis Angelakeris, Constantin M. Boscos

**Affiliations:** 1 School of Veterinary Medicine, Faculty of Health Sciences, Aristotle University of Thessaloniki, 54627 Thessaloniki, Greece; anastasiadousof@yahoo.gr (S.A.); basioura@vet.auth.gr (A.B.); demkaterina@hotmail.com (A.N.); imichos84@hotmail.com (I.M.); anisivet@yahoo.gr (I.K.); tsousis@vet.auth.gr (G.T.); pboscos@vet.auth.gr (C.M.B.); 2 School of Physics, Faculty of Sciences, Aristotle University of Thessaloniki, 54124 Thessaloniki, Greece; theosama@auth.gr (T.S.); ksime@physics.auth.gr (K.S.); agelaker@auth.gr (M.A.)

**Keywords:** ram, sperm, bacterial resistance, microbiological analyses, CASA analyses, nanoparticles, nanotechnology, antibiotics

## Abstract

**Simple Summary:**

Artificial insemination with extended semen is a reproductive biotechnological method promising quick genetic improvement and benefits for farmers. Semen collection in farm animals, including rams, is a procedure that takes place under unsterile conditions. Apart from certain bacteria, which are pathogenic and may be transmitted through semen to the female genital tract, causing clinical diseases, semen from healthy donors may also be contaminated during processing of the ejaculate. Consequently, ejaculate contamination with bacteria is inevitable. Antibiotics are a main constituent of semen extenders to avoid growth of bacteria present in ram semen and, subsequently, enhance reproductive performance. During the last decades, however, there is an increasing scientific interest about alternative agents to the conventional antibiotics, such as nanoparticles, to overcome bacterial resistance, which is a serious problem for human and veterinary medicine. Therefore, the aim of this study was to examine the effect of iron oxide and silver nanoparticles’ supplementation on bacterial proliferation control during ram semen processing, as well as their impact on sperm quality and functionality variables.

**Abstract:**

The aim of the study was to investigate the effect of iron oxide (Fe) and silver (Ag) nanoparticles (NPs) on ram semen. A skim milk extender without antibiotics was used as a diluent of 21 ejaculates (8 rams; 2–3 ejaculates/ram). The groups of control (C; semen without NPs), Fe NPs (3.072 mg Fe_3_O_4_/mL semen), and Ag NPs (2.048 mg Ag-Fe/mL semen) were incubated (15 °C; 30 min), and then a magnetic field was used for NPs’ removal. Standard microbiological procedures were performed for all groups. Post-treated samples were stored (15 °C) for 24 h, and sperm variables (kinetics by computer assisted sperm analysis (CASA); viability; morphology; HOST; DNA integrity) were evaluated at 6 and 24 h. Semen data were analyzed by a mixed model for repeated measures and microbiological data with Student’s *t*-test for paired samples. At 6 h of storage, VCL and rapid movement-spermatozoa, and at 24 h, total/progressive motility and amplitude of lateral head displacement (ALH) were significantly decreased in group Ag compared to control. In group Fe, progressive/rapid movement-spermatozoa were significantly lower compared to control after 24 h of storage. Only in group Ag was a significant reduction of total bacterial count revealed. In conclusion, the examined Fe NPs demonstrated slight antibacterial effect, while the examined Ag NPs provided higher antibacterial properties accompanied by cytotoxicity.

## 1. Introduction

The addition of antibiotics in ram semen extenders is a common method to prevent bacterial contamination and protect the females from microbial transmission, as well as to ensure high quality of semen doses for artificial insemination (AI). Semen bacterial contamination has been associated with a reduction of sperm viability and longevity, high agglutination, low motility, and damage of the acrosomes in different species [1,2,3,4]. The ram semen collection, handling, and processing involves a risk of bacterial contamination of animal (skin, pennis, prepuce) and not-animal (equipment, workers) origin. In sheep AI, the preparation of ram semen doses requires the addition of antibiotics to avoid contamination by usually presented bacteria, such as *Staphylococcus epidermis* and *aureus*, *Proteus mirabilis*, *Escherichia coli*, *Enterobacter cloacae*, and *Pseudomonas aeruginosa* [4]. Nevertheless, the widespread use of antibiotics contributed to the phenomenon of antimicrobial resistance, which is a serious problem for the food industry and human and veterinary medicine. The scientific community has intensified its efforts to find alternative strategies to control bacteria growth and, thus, minimize the risks of developing antibiotic resistance. In this context, the use of colloidal solutions to naturally remove bacteria by centrifugation [5], the supplementation of peptides of natural or synthetic origin in semen extenders [6,7], and the use of natural products with antibacterial activity, such as honey [8], have been reported. Promising results have been published by our scientific team, regarding the use of iron oxide NPs as alternatives to antibiotic additives in boar semen [9].

Nanoparticles (NPs) generally have strong antimicrobial activity. Silver NPs of 40–60 nm have shown significant antibacterial efficacy against *Escherichia coli*, *Pseudomonas aeruginosa*, and *Staphylococcus aureus* [10]. Li et al. [11], while studying the mechanism of action of silver NPs, reported that at concentrations of 10 μg/mL, they inhibit the growth of 10^7^ cfu/mL *Escherichia coli* cells inducing damage to the structure of the cell membrane, constraining the enzymic activity, and causing the death of bacteria. Interesting results were provided by the study of Shahverdi et al. [12], where the antimicrobial effects of vancomycin, amoxicillin, and penicillin against *Staphylococcus aureus* were increased in the presence of silver NPs.

Toxicological screening is a common practice to confirm the potential use of new drugs and chemicals at the clinical level. The toxicity of a substance depends on many factors, such as the gender, the age, the species, and it is mediated by the organism’s ability to absorb and metabolize this substance. The different susceptibility of animal species to various toxic agents has been known for many years, and this situation is fully accepted by the scientific community [13,14,15,16]. The investigation of the potential toxic properties of a new agent in different animal models and the examination of different doses is a common scientific approach, which often reveals diverse results and ensures the safety and the possible wide use of that agent. Regarding NPs, recently Bahamonde et al. [17] have reported that there are important species-specific differences in gold NPs’ biodistribution, excretion, and toxicity potential. Moreover, the NPs’ effects on cells are continuously under research and, according to the literature, these effects on spermatozoa can vary, depending on the type of NPs, as well as on their in vitro or in vivo administration [18].

The present study aimed to investigate, as a first approach, the possible toxic or beneficial effects of iron oxide and silver NPs on the microbial load and the sperm quality and functionality of ram semen samples.

## 2. Materials and Methods

### 2.1. Reagents and Media Preparation

The chemicals and the reagents used in the study were of high analytical grade and supplied by Sigma-Aldrich Chemical Co. (Seelze, Germany) unless otherwise stated.

The media were prepared using sterile conditions in a laminar flow hood (BH 100, Telstar, TELSTAR INDUSTRIAL S.L., Terrassa, Spain). Semen samples were extended in the conventional skim milk extender (100 g powder skim milk/L distilled water; pH 7.2–7.4; 290–300 mosmol/kg) without antibiotics. The EDTA-free phosphate-buffered saline (PBS: 137 mM NaCl, 2.7 mM KCl, 1.5 mM KH_2_PO_4_, 8.1 mM Na_2_HPO_4_; pH 6.8–6.9; 280–300 mosmol/kg) without antibiotics was used for re-extension of sperm samples prior to semen samples analysis.

### 2.2. Nanoparticles

#### 2.2.1. Synthesis of Fe_3_O_4_ and Ag/Fe Nanoparticles

Tested NPs were separately developed to consist of (i) a nanocomposite of silver (Ag) NPs, known for their antimicrobial effect, attached on magnetic responsive zero-valent iron (Fe) NPs, and (ii) single-phase magnetite (Fe_3_O_4_) NPs, which combine magnetic and antimicrobial properties. Single Fe_3_O_4_ NPs were produced by the precipitation of FeSO_4_ in water under mild oxidative conditions defined by the addition of NaNO_3_ [19]. A volume of 100 mL of a 0.02 M FeSO_4_∙7H_2_O solution was mixed with 50 mL of another solution with 0.05 M NaOH, 0.02 M NaNO_3_, and 30 mL ethanol. Immediately, a jelly precipitate of green rust was formed, which gradually turned into a black dispersion after ageing at 90 °C for 24 h under slow stirring. The product was washed with distilled water and centrifuged to receive the purified NPs as a dried product or aqueous dispersion.

For the preparation of the Ag/Fe nanocomposite, the solar-powered physical vapor deposition (SPVD) was applied using the facilities of PROMES institute in Odeillo, France [20]. Particularly, in a Heliotron 2 kW glass vacuum chamber a pellet of pressed Fe and Ag powder (5% wt. Fe) was placed inside and then evaporated by a concentrated solar beam at a pressure of 70 torr controlled under Ar flow. The composite system was recovered after having been trapped on a nanoporous ceramic filter located in the direction of pumping flow.

#### 2.2.2. Characterization

Obtained NPs were studied for their structural phases using a Rigaku Ultima+ X-ray diffractometer (XRD) working with 40 kV/30 mA CuKa radiation, at 0.05° for step in size and 3 s for step in time. For the identification of existing crystal compounds, the diagrams were compared to the ICDD-JCPDS Powder Diffraction Files database. High magnification images of the nanosized samples, which reveal their size and morphology, were obtained by Field-Emission Scanning Electron Microscopy (FE-SEM) using a FEI Quanta 200 ESEM FEG instrument with the field-emission gun adjusted at 30 kV.

#### 2.2.3. Preparation of Fe_3_O_4_ and Ag/Fe NPs Solution

The NPs were dissolved in distilled water to prepare the two stock NPs solutions (Fe_3_O_4_ stock solution: 19.2 mg per mL; Ag/Fe nanocomposite stock solution: 12.8 mg per mL), which were prepared every week and were sonicated for 20 min (to enhance the dispersion stability) before their use.

### 2.3. Animals and Semen Samples Preparation

Semen samples were acquired from eight fertile and healthy adult (2–3 years of age) Lacaune rams, owned by a commercial AI center. The animals were housed in individual pens with uniform feeding and lighting conditions and were routinely used for semen production. Ejaculates (*n* = 21; 2–3 ejaculates/ram) were collected (May–July) using an artificial vagina and were extended 1:5 (v:v) in the skim milk extender. All collected samples fulfilled the quality criteria for commercial semen preparation for AI (total sperm motility ≥80%; sperm concentration ≥3 × 10^9^ sperm/mL; and at least 80% spermatozoa with normal morphology). The diluted semen samples were transferred within 30 min (15 °C) to the Unit of Biotechnology of Reproduction, Clinic of Farm Animals, Faculty of Veterinary Medicine, Aristotle University of Thessaloniki (Thessaloniki, Greece). Upon delivery, the semen samples were re-evaluated for sperm motility and were stored at 15 °C before further processing. All procedures with the animals in this study were conducted in ways consistent with the international guidelines (EU Directive, 2010/63/EU) and according to the rules of the Bioethics Committee of Aristotle University (project’s code: MIS 5004681).

### 2.4. Assessment of Sperm Variables

#### 2.4.1. Sperm Motility and Kinetics

Sperm motility and kinetics were evaluated using a computer assisted sperm analysis (CASA) system (Sperm Class Analyser^®^, Microptic S.L., Automatic Diagnostic Systems, Barcelona, Spain) and a microscope (AXIO Scope A1, Zeiss, Germany) equipped with a heating stage and a camera (Basler scA780 54fc, Germany). The analysis was performed by Sperm Class Analyser^®^ software (SCA^®^ v.6.3.; Microptic S.L., Automatic Diagnostic Systems, Barcelona, Spain) with the following configurations: 4–6 fields were analyzed (×100) for each semen sample, >500 sperm, 25 frames/s, progressive movement of >80% of the parameter STR, depth of field 10, and temperature of the heating stage 37 °C. The debris incorrectly identified as spermatozoa were manually removed before the final analysis.

For each semen sample, an aliquot was further diluted (1:30, *v/v*) to avoid high concentration and to ensure a valid analysis. Afterwards, a volume of 10 μL was placed on the preheated (37 °C) Makler chamber (10 μm deep; Makler^®^ counting chamber, Sefi Medical Instruments, Haifa, Israel) and the following CASA motility and kinetic parameters were evaluated: (1) Total motility; %, (2) progressive and non-progressive movement spermatozoa; %, (3) rapid, medium, and slow movement spermatozoa (10 < slow < 45 < medium < 75 < rapid μm/s); %, (4) curvilinear velocity (VCL); μm/s, (5) straight line velocity (VSL); μm/s, (6) average path velocity (VAP); μm/s, (7) amplitude of lateral head displacement (ALH); μm, (8) beat/cross-frequency (BCF); Hz, (9) linearity (LIN); VSL/VCL × 100, (10) straightness (STR); VSL/VAP × 100 and (11) wobble (WOB); VAP/VCL × 100.

#### 2.4.2. Sperm Viability

Sperm viability was assessed using the double staining eosin-nigrosine in one step [21]. By means of a Zeiss optical microscope (Oberkochen, Germany) at magnification ×1000, 200 spermatozoa were estimated, counted, and the results were expressed as percentage of live spermatozoa in terms of plasma membrane integrity.

#### 2.4.3. Sperm Morphology

Sperm morphology was evaluated by applying the SpermBlue staining protocol (SpermBlue^®^ 08029, Microptic S.L., Barcelona, Spain) following the manufacturer’s instructions [9]. Spermatozoa were assessed microscopically (×400) and classified as normal or with morphological abnormalities [head (including integrity of acrosome membrane), tail, cytoplasmic droplets]. In total, 200 spermatozoa per slide were counted, and the results were expressed in % ratio.

#### 2.4.4. Sperm Membrane Functionality

Sperm membranes functionality was assessed by Hypo-Osmotic Swelling Test (HOST). The HOST analysis was based on Jeyendran et al.’s [22] protocol after applying a slight modification. Briefly, a volume of 100 μL of semen sample was mixed with 1 mL of HOST solution (75 mmol/L fructose, 32 mmol/L sodium citrate; 150 mosmol/kg) and incubated for 1 h at 37 °C. In total, 200 spermatozoa per slide were evaluated (×400). The results were expressed as spermatozoa with swollen tails (%).

#### 2.4.5. Sperm DNA Integrity

Sperm DNA integrity was estimated applying the Acridine Orange Test (AOT), which determine the susceptibility of the DNA to denaturation, detecting single-strand DNA breaks [23]. In total, 200 spermatozoa were counted using a Zeiss fluorescence microscope (×1000; Oberkochen, Germany). The results were reported as percentage (%) of spermatozoa with DNA fragmentation.

### 2.5. Microbiological

Antimicrobial survey was conducted on samples from all experimental groups [control ©, Fe_3_O_4_ (Fe) and Ag/Fe (Ag)] using standard protocols. All used media were supplied by OXOID (Thermo Fisher Specialty Diagnostics Ltd., Basingstoke, UK) and were prepared according to manufacturers’ recommendations. Samples were subjected to tenfold dilutions in 0.9% normal saline before testing. One hundred (100) μL of each dilution were plated into Plate Count agar, and the results were recorded as cfu/mL after 48 h of incubation at 37 °C. The survey focused on the detection of the microbial load and frequently isolated bacteria in ram semen. For this purpose, 100 μL from each dilution were spread on plates containing sheep blood agar, MacConkey agar, Baird Parker medium with egg yolk tellurite emulsion, Kanamycin Aesculin Azide agar, and incubated at 37 °C for the detection of *Staphylococcus* spp., *Streptococcus* spp., *Enterococcus* spp., *Enterobacter* spp., *Bacillus* spp., *Proteus* spp., and *Escherichia coli*. Pseudomonas agar base plates containing CN selective supplement (OXOID, SR0102) were incubated at 25 °C for the detection of *Pseudomonas* spp. Plates were monitored during incubation, and bacterial growth was recorded after 24 and 48 h to include bacteria that may recover, and to obtain an overview of bacterial growth. Bacterial isolates were identified using standard microbiological procedures, taking into account production of haemolysin, culture and colonial characteristics, Gram staining, oxidase- and catalase- reaction, coagulase testing, and other conventional biochemical tests when needed.

### 2.6. Experimental Design

Upon delivery at the Unit of Biotechnology of Reproduction, each semen sample was divided into 3 aliquots, and the following three experimental groups were prepared: (1) Control group (C): Extended semen without any treatment; (2) Fe_3_O_4_ group (Fe): Extended semen with Fe_3_O_4_ NPs (3.072 mg Fe_3_O_4_ per mL of semen); and (3) Ag/Fe group (Ag): Extended semen with Ag/Fe NPs (2.048 mg Ag/Fe per mL of semen).

#### 2.6.1. Pretrial: Determination of the Co-Incubation Time of Semen with NPs

Dose and time are fundamental variables of toxicity. A pretrial was performed to evaluate the beneficial/detrimental NPs’ concentration and co-incubation period of semen samples with NPs. This is a before used valid scientific approach regarding the interaction and the consequences of increasing doses of NPs on major sperm kinetic variables of ram sperm [18]. Considering that collection of ram semen has a higher contamination risk compared to boar semen collection, as well as the previously used doses of Fe_3_O_4_ (0.192 mg per mL) and Ag/Fe NPs (0.128 mg per mL) on boar semen [9,24], multiplied (×2, ×4, ×8, ×16, ×32) doses of NPs were tested. Eight ejaculates (*n* = 8; 1 ejaculate/ram) were used in pretrial. All experimental groups were incubated at 15 °C until 60 min. Nanoparticles were co-incubated for 30, 45, and 60 min. After NPs’ removal (through a magnetic field, as it is described in the main experiment), progressive motility, which is the most important semen variable for fertilization, was assessed for all experimental groups using the CASA system.

#### 2.6.2. Main Trial: Investigation of the Effect of NPs on Ram Semen Quality

The NPs groups were incubated at 15 °C for 30 min after NPs supplementation to treated groups. The same incubation time was employed for the C group, where no NPs were added. Then, NPs were removed by placing the tubes in a plastic rack equipped with commercial NdFeB permanent magnets. The tubes remained in vertical position for 5 min to separate the NPs from semen. The post treated semen samples were carefully collected and placed in a new tube, while the NPs were discarded. This procedure was repeated three times to remove completely the NPs. Finally, the control and the NPs post treated samples were stored at 15 °C for 24 h. The semen variables were assessed 6 and 24 h post treatment. This corresponds to 6.5 and 24.5 h, respectively, from the dilution of collected semen including the transfer time of the semen to the laboratory (30 min). These time points represent the proposed storage time to perform sheep AI with cooled ram semen [25], as well as the market instructions for ram semen storage after dilution with conventional extenders. At 0 h, an aliquot of all the experimental groups was transported (15 °C) within 15 min to the microbiological lab to conduct the antimicrobial survey.

### 2.7. Statistical Analysis

The Statistical Analysis Systems version 9.3 (SAS Institute Inc., 1996, Cary, NC, USA) was used to conduct the statistical analysis. The distribution of the data was analyzed using the Shapiro–Wilk Test (PROC UNIVARIATE). All parameters followed a normal distribution except for the parameters Head and Cytoplasmic droplets. These parameters were square root transformed to achieve normalization. For better clarity, the data are presented as LSmeans and SEM before normalization. The analysis was performed with a mixed model for repeated measures (PROC MIXED). In this model, fixed effects were group, time, and their interaction, and random effect was ram. Semen sample was defined as the subject of the observations. Akaike information criterion (AIC) values were analyzed to opt for covariance structure. Six different structures were modelled (variance components, unstructured, compound symmetry, first order ante dependence, first order autoregressive, and Toeplitz) and the one showing the least AIC was chosen. Pairwise comparisons were conducted based on the Tukey adjustment. The same analysis was used for the pretrial, including time and dose as fixed effects. For the analysis of the microbiological data, differences between control, group Ag, and group Fe were paired for every variable and time point, and their distribution was tested using the Shapiro–Wilk Test. Normality was evident in all cases. A *t*-test for paired observations was used to examine the null hypothesis that the true mean was zero. Microbiological data are presented as means ± SEM. Statistically significant difference was defined as *p* < 0.05.

## 3. Results

### 3.1. Nanoparticles’ Validation

**Figure 1:**
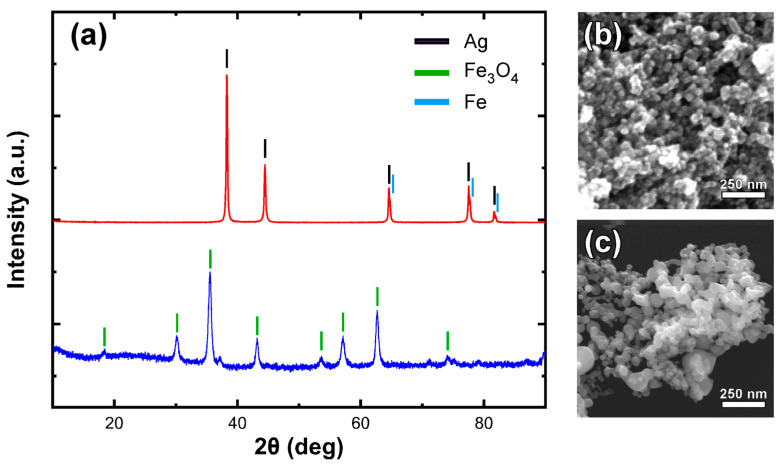
XRD diagram (Intensity versus 2theta angle) of used Fe_3_O_4_ or Ag/Fe nanoparticles (**a**), and corresponding FE-SEM observation images for Fe_3_O_4_ (**b**) and Ag/Fe nanoparticles (**c**).

The composition and morphological characteristics of the tested nanoparticles were validated by means of XRD and FE-SEM analysis. Figure 1 shows the identification of Fe_3_O_4_ and Ag as the major phases in each nanoparticle system. Due to the similarity of the expected peaks for Ag and Fe, the presence of the small percentage of iron in the second sample is only indicated by the side peaks observed at high-angle peaks. Electron microscopy images (Figure 1b,c) demonstrate the presence of spherical nanoparticles in both cases with an average size range around 30–40 nm.

### 3.2. Results of the Pretrial

**Figure 2:**
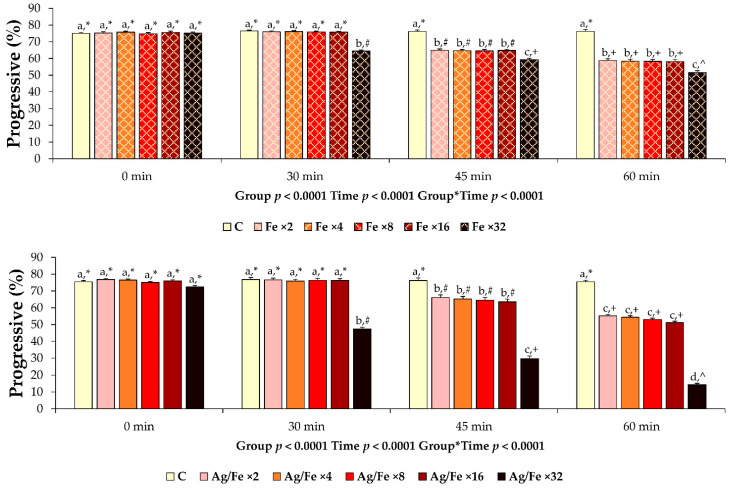
Post treatment progressive motility of liquid extended ram semen samples (*n* = 8) co-incubated (15 °C) with different concentrations of Fe_3_O_4_ and Ag/Fe nanoparticles (NPs) for 0, 30, 45, or 60 min. Control group (C): Extended ram semen samples-no treatment; iron oxide groups (Fe): Extended ram semen supplemented with different concentrations of Fe_3_O_4_ NPs (Fe ×2, Fe ×4, Fe ×8, Fe ×16, Fe ×32 correspond to 0.384 mg, 0.768 mg, 1.536 mg, 3.072 mg, and 6.144 mg of Fe_3_O_4_ NPs/mL semen, respectively); silver groups (Ag/Fe): Extended ram semen samples supplemented with different concentrations of Ag/Fe NPs (Ag/Fe ×2, Ag/Fe ×4, Ag/Fe ×8, Ag/Fe ×16, Ag/Fe ×32 corresponds to 0.256 mg, 0.512 mg, 1.024 mg, 2.048 mg and 4.096 mg of Ag/Fe NPs/mL semen, respectively). Values are expressed as LSmeans ± standard error of the mean (SEM). Differences at statistical level between groups are noticed by different superscripts (a, b, c, d). Significant differences between evaluation times within each experimental group are depicted by different symbols (*, #, +, ^).

The results revealed cytotoxicity in a time/dose dependent manner (Figure 2). The co-incubation period of 45 and 60 min were excluded due to a significant deterioration of the evaluated sperm motility characteristic compared to the control group. The co-incubation period of 30 min showed no adverse effects compared to control and was selected for further research. The higher concentrations of NPs (×16 for Fe_3_O_4_; ×16 for Ag/Fe), which did not affect progressive motility at 30 min of co-incubation, were selected for the main trial (3.072 mg Fe_3_O_4_ per mL of semen; 2.048 mg Ag/Fe per mL of semen).

### 3.3. Results of the Main Trial

**Figure 3:**
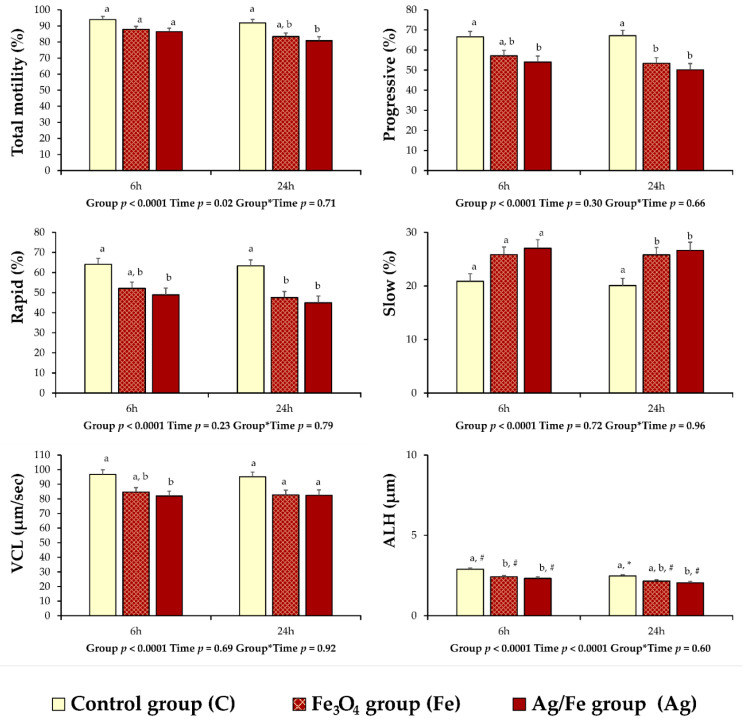
CASA (computer assisted sperm analysis) motility and kinetics variables of liquid extended ram semen (*n* = 21) preserved at 15 °C for 6 and 24 h post treatment with Fe_3_O_4_ or Ag/Fe nanoparticles (NPs). Control group (C): Extended ram semen samples-no treatment; iron oxide group (Fe): Extended ram semen with Fe_3_O_4_ NPs (3.072 mg Fe_3_O_4_ per mL of semen); silver group (Ag): Extended ram semen with Ag/Fe NPs (2.048 mg Ag/Fe per mL of semen). Values are expressed as LSmeans ± standard error of the mean (SEM). Differences at statistical level between groups are noticed by different superscripts (a, b). Significant differences between evaluation times within each experimental group are depicted by different symbols (#, *).

According to the results of the CASA motility variables, differences were observed between the control and the treated groups. At 6 h of storage at 15 °C, the values of progressive (*p* = 0.027) and rapid (*p* = 0.017) movement spermatozoa, VCL (*p* = 0.018), and ALH (*p* = 0.0003) of group Ag were lower compared to group C (Figure 3). At the same time point, a similar situation was noticed for ALH (*p* = 0.003) between the Fe and C groups (Figure 3). In group Ag, the values of total motility (*p* = 0.015) and ALH (*p* = 0.006) were less compared to group C at 24 h of storage (Figure 3). In both NPs treated groups, decreased values of progressive and rapid movement spermatozoa (*p* < 0.05) were recorded after 24 h of storage compared to group C, while the values of slow movement spermatozoa (*p* < 0.05) were increased (Figure 3).

There was an overall group effect regarding the parameters of non-progressive movement spermatozoa, VAP, STR, and BCF (Table 1). However, these differences were not so pronounced and pairwise comparison after adjustment revealed no pairwise statistical effects between groups in the discrete time points. For the remaining CASA variables, which are medium movement spermatozoa, VSL, LIN, and WOB, there were no statistical differences (*p* > 0.05) between groups (Table 1). An effect of time was noticed in group C and higher values for LIN (*p* = 0.007), STR (*p* = 0.02) and WOB (*p* = 0.02) were noticed at 24 h of storage compared to 6 h (Table 1), while the opposite result was obtained for ALH (*p* = 0.006; Figure 3). Regarding the variables of viability, in spermatozoa with cytoplasmic droplets and HOST positive spermatozoa (Table 2), no group but an overall time effect was present. However, once again, pairwise comparisons within groups and between time points were not significant. No group or time effect was evident for head abnormalities.

Spermatozoa with normal morphology were lower in group Ag compared to group C at 6 (*p* = 0.002) and 24 h (*p* < 0.0001) of storage (64.94 ± 2.56 vs. 78.47 ± 2.30 and 62.56 ± 2.78 vs. 80.33 ± 2.43 for 6 and 24 h, respectively). According to the statistical analysis for each category of the sperm morphological abnormalities, the deterioration of these parameters corresponds only to the percentage of spermatozoa with abnormal tail which was higher in group Ag (*p* = 0.007 and *p* = 0.003 for 6 and 24 h, respectively), compared to control (33.05 ± 2.75 vs. 19.80 ± 2.47 and 34.00 ± 3.00 vs. 18.76 ± 2.62 for 6 and 24 h, respectively).

The values of DNA fragmentation in control samples were low without clinical relevance, between 1–2%, while the treatment of semen samples with the examined NPs did not affect this sperm variable.

Microbial growth was observed in all semen samples, but the isolated bacteria and overall bacterial load varied significantly among them. The total bacterial count was rather low, reaching up to 1.8 × 10^4^ cfu/mL in diluted semen on the first day of the trial. Isolated bacteria on samples belonged predominantly to the *Staphylococcus* species, followed by *Enterococcus* spp. and Enterobacteriaceae, while bacterial presence in control and experimental groups varied substantially (Table 3).

Culture on selective media demonstrated the antimicrobial activity of both Ag and Fe NPs to be depended on the microbial strain. The bacterial examination of the semen revealed that the microbial reduction for some samples was intense, while on others had a minor effect (Table 3). Concerning the two NPs treated groups, Ag group revealed a statistically significant reduction for total bacterial count after 48 h of incubation at 37 °C (*p* = 0.03), while Fe group expressed antibacterial action, but it did not have a repetitive result on bacteria inactivation.

## 4. Discussion

In view of the recent criticism on synthetic drugs and the increase of antibiotic resistance, a trend to replace conventional antimicrobials using alternative substances evolves. Nanoparticles express antimicrobial activity against different microorganisms, making them a promising novel approach to control bacterial infections [26]. However, toxic effects of NPs on cells and tissues have been reported [27].

In the present study, silver or iron oxide NPs were co-incubated for 30 min without detrimental effects to sperm motility. In the pretrial, the co-incubation time of 45 and 60 min adversely affected the movement of spermatozoa in higher than the selected concentrations for the main trial. Although time and dose are critical factors for NPs’ toxicity, it is considered that many other factors, such as size, shape, surface, stability, physicochemical properties, magnetic activity, and thermal and electrical conductivity of NPs can affect the dynamic of toxicity, contributing to this result [28]. After that, the NPs were removed, and sperm quality variables and microbial load were evaluated at times relevant (6 to 24 h) to usual ram semen storage for field AI [25].

Ram fertilizing ability depends on sperm quality, highlighting motility as the most accredited parameter in sheep AI centers. Computer-assisted sperm analysis supports an objective sperm motility and kinetics’ evaluation [29]. In our study, the results showed a deterioration of ram semen handled with Ag/Fe NPs after 6 to 24h. Specifically, the group of Ag/Fe NPs showed lower values of total motility and normal morphology and higher values of sperm tail abnormalities at 24 h, as well as lower rapid movement spermatozoa and VCL at 6 h of storage compared to the control group. It is noteworthy that lower values of progressive and rapid movement spermatozoa were evident only after 24 h, yet not at 6 h, in Fe group. These results could indicate a higher toxic effect of Ag/Fe compared to iron oxide NPs on ram spermatozoa. Nevertheless, both NP groups succeeded in preserving satisfactory values regarding the main variables investigated at 6h of storage, with the iron oxide group being superior to Ag/Fe group. Taking into consideration that in sheep practice AI with fresh cooled semen should be performed within 6–8 h after semen extension [25], the use of NPs could be justified. In our previous study, no toxic effect was observed for Fe_3_O_4_ NPs on boar spermatozoa even after 48 h of storage [9]. Among the adverse effects of NPs, differences regarding cells’ susceptibility are acknowledged, thus in vitro tests for Nano safety show particular attention to cell type selection [30]. Falchi et al. [18,31] support that consideration should be given to the specific effects of NPs on male gametes, where cytotoxicity takes place in a time/dose dependent manner, with a species susceptibility to be a potential diversification factor. Moreover, a dose and time-dependent detrimental effect of silver NPs on rat epididymal spermatozoa has been reported [32]. Spermatozoa’s fatty acid and lipid composition vary between species, and between animals of the same species [33]. The sperm plasma membrane plays a crucial role in the regulation of fertilization functions. However, it varies between mammalian species concerning its structure and molecular composition [34]. Lipidic changes have been indicated as factors affecting the maturation in epididymis and as a result the freezability of ram sperm [35]. In addition, the susceptibility of sperm at chilling and freezing has been attributed to differences regarding the architecture, ratio, and distribution of the membrane’s lipids [36]. Different cytotoxicity phenomenon of Ag NPs in boar and ram spermatozoa could also be attributed in differences related to species, yet this issue warrants further investigation.

Ram sperm viability, total and progressive motility, movement of spermatozoa (rapid, medium, slow), normal morphology, sperm membranes functionality, and the CASA evaluated kinetics (VCL, VSL, VAP, STR, LIN, WOB, and BCF) were not affected in iron oxide NPs compared to control considering the time limitation of 6h. These variables are of high importance to semen’s fertilizing capacity, and thus suggest the use of Fe_3_O_4_ NPs for the handling of ram semen. This view is supported by the results of Robayo et al. [29], who reported that ram sperm motility, VCL, and VAP present significant positive correlations with the sperm’s migration ability in sheep cervical mucus. Moreover, Santolaria et al. [37] found that VCL and sperm viability have predictive potential regarding ram field fertility. Vicente–Fiel et al. [38] recorded significantly higher values of VCL, VSL, VAP, LIN, STR, and ALH in adult rams of high compared to rams of low fertility. The relationship between field fertility and CASA variables has been studied more extensively in bull sperm, where progressive motility, rapid movement spermatozoa [39], and VAP [40] have been found to show the greatest influence on fertility.

Concerning sperm DNA fragmentation, low values without clinical relevance, were observed in the present study. It is notable that the performed acridine orange test determines the susceptibility of the DNA to denaturation and detects only single-strand DNA breaks. Perhaps, this sperm parameter must be revaluated in a future study by a method with higher sensitivity, able to detect single- and double-strand DNA breaks. The COMET under neutral pH conditions and the TUNEL assays, which determine real DNA damage can clarify this issue [41].

Furthermore, silver, its compounds and the formed NPs have been tested and proved to be effective against Gram-positive and Gram-negative pathogenic bacteria. Their antibacterial action against *E. coli* [11,42] has a concentration and shape-dependent interaction, whereas against *S. aureus* [43], they affect the genomic DNA sequence. Moreover, their action against multidrug resistant *P. aeruginosa* is time and concentration dependent [44]. Other researchers report findings that suggest bacterial resistance to NPs for some microorganisms [45,46,47]. Alternatively, iron NPs gain ground for their characteristics and antibacterial properties, as they inhibit *S. aureus* [48], express antibacterial activity against *E. coli* [49], and suppress the growth of *P. aeruginosa* [50].

In the present study, Ag/Fe and Fe_3_O_4_ NPs were examined as a potential solution to reduce or prevent bacteria growth in ram semen. Contaminants were present in all collected samples, however, the microbial load varied among them. Total bacterial count for aerobic mesophiles was up to 1.8 × 10^4^ in diluted semen after ejaculation, while Yániz et al. [4] reported concentrations up to 10^8^ cfu/mL in commercial ram semen. The predominant isolated bacteria belonged to *Staphylococcus* spp., *Enterococcus* spp., and Enterobacteriaceae, which is in accordance with previously reported studies in rams [4].

The antimicrobial profile of silver and iron oxide NPs varied among the examined samples. In some samples silver NPs managed to reduce the load of *Staphylococcus* and *Enterococcus* strains, however their antibacterial action depended on strain sensitivity. Despite the different degrees of contamination among samples, a significant reduction for the total bacteria count was observed in the presence of Ag/Fe NPs after 48 h compared to control. A recent study of Pérez-Duran et al. [51] describes the inhibition of *S. aureus* in swine semen using a low concentration of Ag/Fe NPs without any toxic effect on semen.

The application of iron oxide NPs on ram semen tended to prevent *Staphylococcus* spp., although this effect did not reach statistical significance. Their activity appeared to be preventive for *Staphylococcus* spp., *Enterococcus* spp., and Enterobacteriaceae, and in some samples the microbial reduction was intense; however, the bacterial counts ranged among samples, indicating a non-repetitive result. These findings are not aligned with the reduction of the bacterial load previously described by our research team [9], regarding the antibacterial action of iron NPs on boar semen. Further investigation is necessary to clarify the factors that suppressed the antimicrobial properties of the NPs in the present study.

The advantageous properties of NPs regarding the amelioration of the antimicrobial activity of antibiotics and the inhibition of biofilm formation, as systems for drug delivery in multiple biomedical applications, have been widely reported [26,52,53]. Despite that, reports discuss their gains and risks, investigating their potential toxic effects in different organs and the reproductive system [54,55]. Silver NPs were found to increase expressed toxicity in rat and mouse semen, but not in human sperm [56,57]. Iron NPs on boar semen controlled bacterial contamination without impairing sperm characteristics [9] and showed no toxicity when used after nano selection of spermatozoa and enrichment of semen for AI [58]. It is known that NPs express antibacterial activity depending on the size, the concentration and the duration of contact interaction with the target microorganisms [59,60]. It is suggested that exploitation of NPs on animal assisted reproductive management could enhance fertility, considering that different types of NPs produce different effects on semen [18,61]. In our study, Ag/Fe NPs demonstrated higher antimicrobial capacity, along with higher cytotoxicity on ram sperm. On the other hand, Fe_3_O_4_ NPs showed no toxic effects on spermatozoa, yet minor antibacterial efficacy. Probably this phenomenon is due to the different properties of the two types of NPs. Magnetite NPs can generate reactive oxygen species (ROS) through an equilibrium of dissolved Fe ions and the production of free radicals (OH˙, HO_2_˙) following Fenton-like reactions [62]. Bacteria corruption can be caused by membrane electrostatic modification due to free radicals. It is probable that the recorded stronger effect of Ag compared to Fe_3_O_4_ NPs on both bacterial and spermatozoa’s membranes, can be attributed to higher generation of ROS and free radicals compared to Fe_3_O_4_ NPs. It is well known that the hyperproduction of ROS exceeds the antioxidant capacity of the seminal plasma, results in oxidative stress and negatively affects semen quality [63]. This is also supported by the high susceptibility of ram spermatozoa to lipid peroxidation, as their membranes are rich in polyunsaturated fatty acids [64]. Future studies with the use of a balanced mix of different concentrations and types of NPs in combination with antioxidants could help clarify this issue.

## 5. Conclusions

In conclusion, the use of NPs could open a new perspective to prevent or reduce bacterial infection in ram semen. The examined Fe_3_O_4_ NPs were not harmful for ram spermatozoa, but they did not satisfy the antibacterial purposes of their use. On the other hand, the examined Ag/Fe NPs revealed higher antibacterial properties than Fe_3_O_4_ NPs along with higher cytotoxicity. Further studies need to demonstrate their use as antimicrobials, accounting for different parameters that interact with infectious bacteria (e.g., size, concentration, time, combination with other NPs or antibiotics in lower concentration).

## Figures and Tables

**Table 1:** Computer-assisted sperm analyzer (CASA) kinetics (LSmeans ± SEM) of liquid ram semen (*n* = 21) preserved at 15 °C for 6 h and 24 h post treatment with Fe_3_O_4_ and Ag_/_Fe nanoparticles (NPs).

Variable	6 h			24 h			*p* Value		
	Group C	Group Fe	Group Ag	Group C	Group Fe	Group Ag	Group	Time	G*T
Non progr. (%)	27.38 ± 1.57	30.20 ± 1.57	32.39 ± 1.75	24.75 ± 1.38	30.02 ± 1.41	30.65 ± 1.58	0.0017	0.23	0.70
Medium (%)	9.04 ± 0.80	9.87 ± 0.80	10.50 ± 0.89	8.47 ± 0.70	10.03 ± 0.71	9.24 ± 0.80	0.22	0.39	0.67
VSL (μm/s)	59.84 ± 2.69	57.79 ± 2.69	57.62 ± 2.99	66.61 ± 3.06	61.07 ± 3.14	62.67 ± 3.51	0.39	0.50	0.83
VAP (μm/s)	77.76 ± 3.08	70.33 ± 3.08	69.45 ± 3.43	81.06 ± 3.29	70.74 ± 3.37	71.77 ± 3.77	0.0092	0.46	0.90
LIN (%)	55.56 ± 1.45 ^#^	59.37 ± 1.45	60.57 ± 1.61	62.75 ± 1.39 *	63.43 ± 1.42	63.82 ± 1.59	0.10	0.0001	0.37
STR (%)	69.44 ± 1.01 ^#^	72.73 ± 1.01	73.57 ± 1.12	73.93 ± 0.94 *	75.73 ± 0.96	75.26 ± 1.08	0.01	0.0004	0.40
WOB (%)	76.14 ± 1.09 ^#^	77.22 ± 1.09	77.85 ± 1.21	80.79 ± 0.95 *	79.13 ± 0.98	79.79 ± 1.09	0.84	0.0017	0.32
BCF (Hz)	6.77 ± 0.13	6.71 ± 0.13	6.33 ± 0.14	6.67 ± 0.12	6.51 ± 0.12	6.16 ± 0.14	0	0.15	0.93

Group C: No treated extended ram semen samples, Group Fe: Extended ram semen samples treated with Fe_3_O_4_ NPs (3.072 mg Fe_3_O_4_ per mL of semen); group Ag: Extended ram semen samples treated with Ag/Fe NPs (2.048 mg Ag/Fe per mL of semen). Experimental time points: 6 and 24 h: Experimental storage period post removal of NPs. Non progr.: Non-progressive movement spermatozoa (%); medium: Medium movement spermatozoa (<25 < medium < 45 μm/s; %); VSL: Straight line velocity (μm/s); VAP: Average path velocity (μm/s); LIN: Linearity (VSL/VCL × 100); STR: Straightness (VSL/VAP × 100); WOB: Wobble (%); BCF: Beat/cross-frequency (Hz). Significant differences between evaluation times within each experimental group are depicted by different symbols (#, *). G*T: Group*Time interaction.

**Table 2:** Sperm quality of liquid ram semen (*n* = 21), (LSmeans ± SEM) preserved at 15 °C for 6 h and 24 h post treatment with Fe_3_O_4_ and Ag/Fe nanoparticles (NPs).

Variable (%)	6 h			24 h			*p* Value		
	Group C	Group Fe	Group Ag	Group C	Group Fe	Group Ag	Group	Time	G*T
Viability	66.85 ± 2.39	68.90 ± 2.39	68.94 ± 2.66	63.28 ± 2.86	63.65 ± 2.93	63.18 ± 3.27	0.89	0.03	0.91
Head abnorm.	0.95 ± 0.35	1.47 ± 0.35	1.35 ± 0.38	0.66 ± 0.27	0.8 ± 0.28	0.93 ± 0.32	0.47	0.06	0.68
Cytopl. dropl.	0.33 ± 0.48	0.52 ± 0.48	1.00 ± 0.53	0.66 ± 0.48	1.7 ± 0.49	2.00 ± 0.55	0.08	0.006	0.27
HOST+	21.90 ± 2.59	29.85 ± 2.59	28.64 ± 2.88	30.28 ± 2.37	32.2 ± 2.43	32.5 ± 2.71	0.10	0.02	0.45

Group C: No treated extended ram semen samples, Group Fe: Extended ram semen samples treated with Fe_3_O_4_ NPs (3.072 mg Fe_3_O_4_ per mL of semen); group Ag: Extended ram semen samples treated with Ag/Fe NPs (2.048 mg Ag/Fe per mL of semen). Experimental time points 6 and 24 h: Experimental storage period post removal of NPs. Viability: Spermatozoa with intact plasma membrane (%); head abnorm: Spermatozoa with head abnormalities (%); cytopl. dropl.: Spermatozoa with cytoplasmic droplets (%); HOST +: Spermatozoa with swollen tails (%). G*T: Group*Time interaction.

**Table 3:** Microorganisms (cfu/mL, means ± SEM) isolated from ram semen samples (*n* = 21) after 24 h and 48 h of incubation at 37 °C on blood agar and selective culture media.

Variable		C	Fe	Ag	Difference (C-Fe)	*p* Value	Difference (C-Ag)	*p* Value
24 h	Bacterial load	1352 ± 896	618 ± 233	657 ± 353	734 ± 329	0.58	695 ± 2537	0.10
	*Staphylococcus* spp.	349 ± 170	320 ± 165	366 ± 178	29 ± 142	0.43	−18 ± 69	0.65
	*Enterococcus* spp.	305 ± 150	193 ± 99	221 ± 109	112 ± 366	0.65	84 ± 311	0.49
	Enterobacteriaceae	261 ± 100	343 ± 112	325 ± 112	−82 ± 207	0.19	−64 ± 174	0.25
48 h	Bacterial load	4528 ± 2596	4098 ± 2082	4125 ± 2373	430 ± 3544	0.94	402 ± 1628	0.03
	*Staphylococcus* spp.	853 ± 358	634 ± 239	761 ± 314	219 ± 819	0.97	93 ± 711	0.59
	*Enterococcus* spp.	1342 ± 903	1350 ± 1000	1400 ± 999	−8 ± 656	0.46	−58 ± 676	0.39
	Enterobacteriaceae	1568 ± 634	2746 ± 1185	2578 ± 1080	−1178 ± 3194	0.25	−1010 ± 2569	0.43

Group C: No treated extended ram semen samples, Group Fe: Extended ram semen samples treated with Fe_3_O_4_ NPs (3.072 mg Fe_3_O_4_ per mL of semen); group Ag: Extended ram semen samples treated with Ag/Fe NPs (2.048 mg Ag/Fe per mL of semen).

## Data Availability

Data sharing not applicable.
